# Reducing rural health inequity through drone-based delivery: a spatial multi-criteria decision framework prioritizing epidemiological vulnerability in hub location

**DOI:** 10.3389/fpubh.2026.1897414

**Published:** 2026-09-14

**Authors:** Jingling Zhong, Yan Zhao

**Affiliations:** 1 Research Center for the Smart Health and Elderly Care Industry in the Guangdong-Hong Kong-Macao Greater Bay Area, Guangzhou Huashang College, Guangzhou, Guangdong, China; 2 School of Management, Guangzhou Huashang College, Guangzhou, Guangdong, China

**Keywords:** aging population, emergency medical services, epidemiological vulnerability, health resource allocation, medical drone delivery, spatial health equity

## Abstract

**Objective:**

To alleviate spatial health inequities in rural areas by optimizing unmanned aerial vehicle (UAV) medical hub location based on an epidemiological vulnerability-driven approach, correcting for the tendency of current logistics-centric models to overlook high-need rural populations.

**Methods:**

A spatial multi-criteria decision-making (MCDM) framework was developed for a simulated rural–urban fringe experiencing severe demographic aging. We defined a novel Epidemiological Vulnerability Index (EVI) incorporating three core dimensions: demographic aging, chronic disease burden (e.g., cardiovascular risks), and baseline healthcare deprivation (ground EMS delay). The framework integrates Geographic Information Systems (GIS) with the enhanced two-step floating catchment area (E2SFCA) method, Analytic Hierarchy Process (AHP), and Technique for Order Preference by Similarity to Ideal Solution (TOPSIS) to rank and allocate highly-prioritized UAV hub sites across 400 grid cells. All results are derived from a calibrated simulation environment and should be interpreted as model-based projections rather than empirical observations.

**Results:**

Simulation results indicated that the “Digital Inverse Care Law” emerged naturally under traditional logistics models, which allocated 85% of hubs to low-vulnerability urban centers. In contrast, the EVI-driven framework strategically anchored 65% of hubs in deep rural pockets. Under the baseline parameter assumptions, this reallocation of UAV hubs reduced the simulated average rural emergency response time by 74.8% (from 45.2 to 11.4 min) in the model. The 11.4-min figure represents UAV delivery time to the incident location, not complete emergency intervention time. This modeled improvement brought an estimated 88.4% of the highly vulnerable aging population into the “Golden 15-Minute” survival window within the simulation.

**Conclusion:**

By embedding epidemiological metrics into spatial planning, this framework provides a decision-support tool that is traceable and reproducible for health policymakers, based on model outputs rather than empirical field validation. It illustrates how targeted digital health infrastructure deployment could, under the model assumptions, contribute to narrowing the rural–urban emergency health divide. These findings are contingent on the specific model assumptions and require field validation and formal health-economic evaluation before real-world implementation.

## 1 Introduction

### 1.1 The persistent rural–urban health divide and spatial accessibility

The rural–urban health divide remains a pervasive global challenge, particularly in Low- and Middle-Income Countries (LMICs) where rapid demographic aging outpaces the development of terrestrial emergency medical infrastructure (1). As global urbanization accelerates, medical resources-including highly trained professionals, advanced diagnostic equipment, and rapid emergency response systems-have concentrated disproportionately in metropolitan centers. Traditional methodologies for assessing healthcare access have increasingly integrated both spatial and nonspatial factors to accurately define health professional shortage areas (2). Among these approaches, the two-step floating catchment area (2SFCA) method has become a gold standard for measuring the spatial accessibility of primary healthcare, capturing the nuanced dynamics of supply-to-demand ratios across varying geographic catchments (3).

However, spatial distance alone does not capture the full spectrum of health disparities. Social vulnerability to environmental and systemic hazards plays a crucial, often overlooked role in determining health outcomes and access to timely medical interventions (4). The temporal and spatial changes in social vulnerability (5), frequently quantified through specialized indices for disaster management (6), reveal that rural health disparities are deeply intertwined with underlying population health profiles and specific rural cultures (7). For instance, rural populations often exhibit higher proportions of older adults, greater burdens of chronic illnesses (such as cardiovascular diseases and diabetes), and lower health literacy. Consequently, transport geographers and public health researchers continually emphasize the imperative to evaluate spatial accessibility strictly through the lens of health equity (8). Enhanced computational models, such as the E2SFCA method, have been developed to better capture these nuanced disparities by incorporating distance decay functions and population-specific needs (9), aligning directly with the public health goal of defining and achieving health equity (10).

### 1.2 The evolution of UAVs in digital public health interventions

In recent years, Unmanned Aerial Vehicles (UAVs), commonly known as drones, have emerged as a digital public health tool that can bypass terrestrial infrastructure deficits. Initial economic evaluations demonstrated the substantial operational value of using drones to transport temperature-sensitive biologics, such as vaccines, in remote regions of sub-Saharan Africa (11). This capability expanded into drone delivery models for medical emergencies, complementing traditional logistics approaches (12). UAVs are now enabling novel surgical and medical applications, such as the rapid transport of antivenom to snakebite victims in mountainous terrains or the delivery of blood products for postpartum hemorrhage (13). Furthermore, during the COVID-19 pandemic, drones played a critical role in delivering healthcare resources and collecting swab samples during regional lockdowns, proving their resilience in times of systemic crisis (14).

For emergency medical services (EMS), time is the most critical determinant of survival. In the context of out-of-hospital cardiac arrests (OHCA), the probability of survival decreases by 7 to 10% for every minute of delay in defibrillation. Drones have proven highly effective in significantly reducing the time to delivery of automated external defibrillators (AEDs) compared to ground vehicles, frequently arriving minutes before traditional ambulances (15). Optimizing drone networks to deliver AEDs represents a structural reallocation in pre-hospital emergency care, extending the “golden window” of resuscitation into previously inaccessible rural areas (16). Similarly, drone-aided healthcare services for patients with mobility-limiting chronic diseases in rural areas have shown substantial clinical potential, ensuring uninterrupted supply chains for essential medications (17). UAVs are now widely recognized as reliable tools for general medical product transport (18), particularly for improving fragmented health supply chains in low-resource settings (19) and facilitating the routine, time-sensitive transport of laboratory specimens (20).

To visualize the structural superiority of UAVs in overcoming terrestrial constraints, Figure 1 illustrates the conceptual framework of our spatial accessibility model. It contrasts the rigid, infrastructure-dependent response of traditional ground EMS with the flexible, topography-agnostic delivery modality of UAV networks, highlighting their pivotal role in securing the ‘Golden 15-Minute’ survival window for remote aging communities.

**Figure 1:**
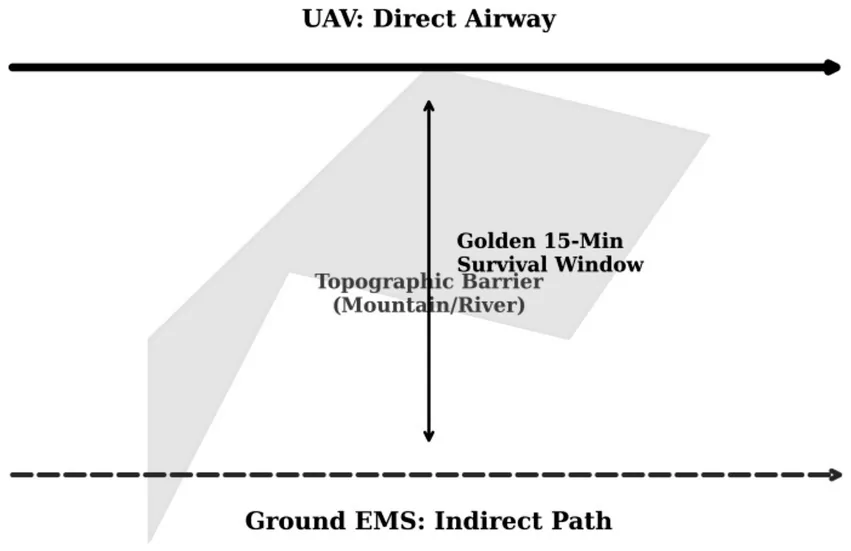
Conceptual comparison of spatial accessibility and response times between traditional ground Emergency Medical Services (EMS) and Unmanned Aerial Vehicle (UAV) delivery networks in rural topographies.

### 1.3 Theoretical conflict: commercial logistics vs. public health equity

Despite these technological and clinical advances, the current deployment of UAV healthcare infrastructure has a notable gap: hub location models are predominantly driven by commercial logistics efficiency rather than epidemiological needs. Conventional operations research models for facility siting—such as the p-median problem, location-allocation algorithms, and the Maximum Coverage Location Problem (MCLP)—have historically focused on minimizing aggregate distance and maximizing raw population coverage (21–23). When applied to drone networks, these traditional algorithmic objectives inherently favor densely populated urban and peri-urban centers, where a single UAV hub can mathematically cover the maximum number of people at the lowest per-flight cost.

While Geographic Information Systems (GIS) and Multi-Criteria Decision Making (MCDM) methods have been widely combined for evaluating industrial locations, such as photovoltaic farms (24), their robust, standardized application in healthcare decision-making is still maturing (25). In the absence of epidemiological guidance, the algorithmic deployment of UAV networks risks exacerbating what we term the “Digital Inverse Care Law.” Originally formulated by Julian Tudor Hart in 1971, the Inverse Care Law states that the availability of good medical care tends to vary inversely with the need for it in the population served. In the digital age, this pattern is reinforced by optimization algorithms: advanced medical infrastructure (like UAV hubs) disproportionately aggregates in wealthy, low-risk urban areas (driven by cost-efficiency optimization), while actively bypassing highly vulnerable rural populations whose geographic dispersion and lower economic density yield lower algorithmic returns on investment.

As illustrated in Figure 2, where the light gray and dark gray bars in the legend, respectively, denote the logistics-driven and health-equity hub allocations, the ‘Digital Inverse Care Law’ creates a paradoxical spatial distribution of resources. While logistics-driven models centralize high-tech hubs in affluent urban cores, our proposed health-equity framework indicates a strategic reallocation toward rural pockets with high epidemiological vulnerability.

**Figure 2:**
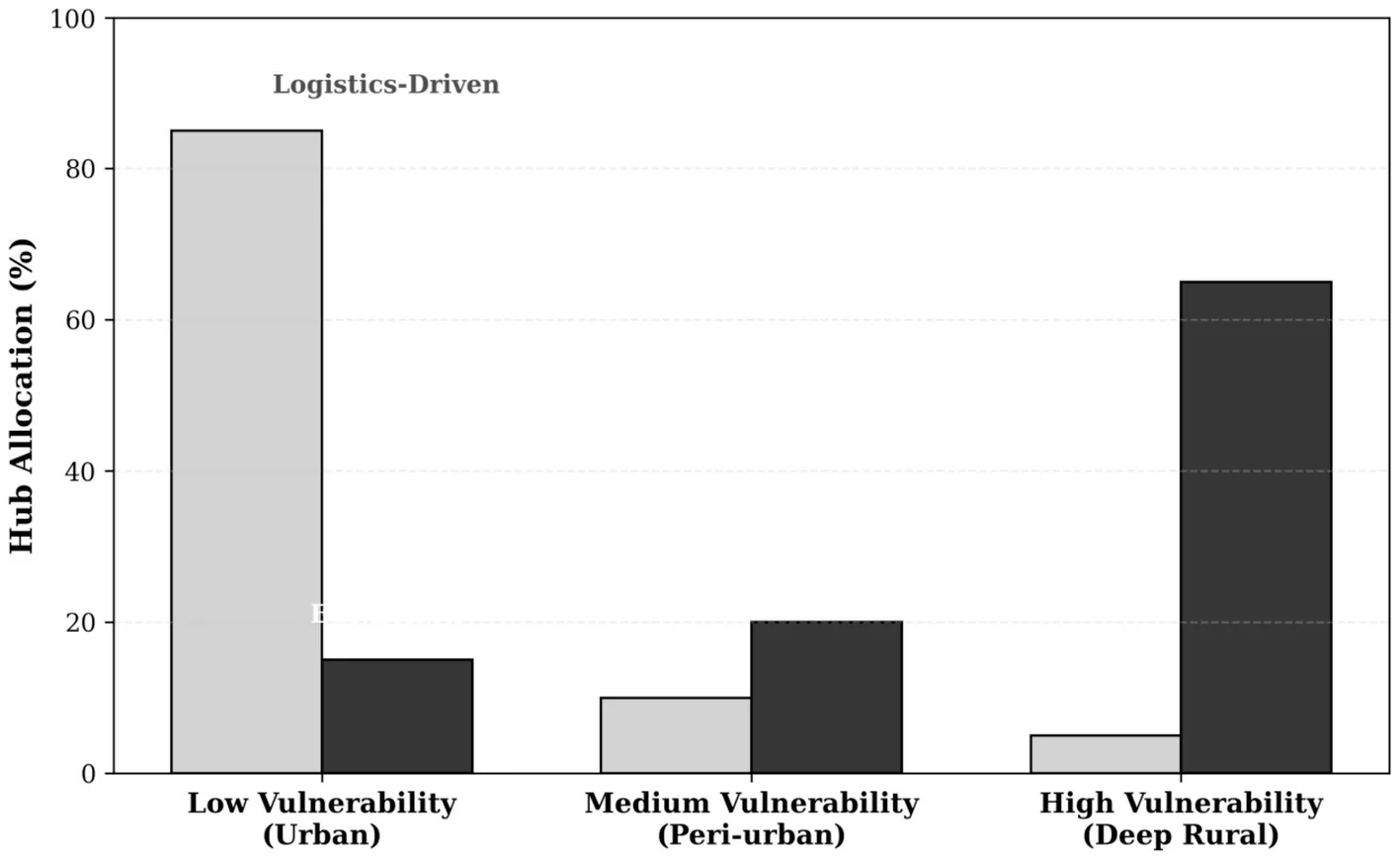
The manifestation of the “Digital Inverse Care Law” in UAV hub allocation. Light gray bars represent the logistics-driven allocation; dark gray bars represent the proposed health-equity allocation. Logistics-driven models concentrate resources in low-vulnerability urban areas, whereas health-equity models redistribute them to high-vulnerability rural pockets.

### 1.4 Research gap, objectives, and contributions

To reduce health inequities, public health infrastructure planning should incorporate equity measurement frameworks alongside engineering efficiency. Extensive GIS-based MCDA literature (26) and recent EMS location optimizations (27) highlight this necessity. While advanced drone network clustering (28), hospital network planning under uncertainty (29), and foundational equity measurements (30) have informed facility location methodologies, previous location-allocation modeling in developing nations (31) and advanced multiobjective optimization approaches (32) indicate a much-needed structural reallocation towards balancing efficiency and equity. However, modern drone networks have not yet fully internalized this public health logic. There is an urgent need for a spatial decision-support system that integrates population health profiles directly into the core prioritization criteria of UAV hub siting.

Recent literature (2024–2026) has increasingly recognized the necessity for advanced decision-making frameworks in healthcare logistics. For instance, recent studies have explored complex emergency medical facility site selection in drone-based relief operations using methods such as the enhanced T-spherical fuzzy Frank combined compromise solution method (33–37). While these advanced MCDM techniques significantly improve operational resilience under uncertainty, current macroeconomic drone network deployments have yet to fully internalize spatial epidemiological metrics as primary objective functions.

To bridge this critical gap, this study proposes a novel spatial Multi-Criteria Decision-Making (MCDM) framework that actively prioritizes “epidemiological vulnerability” in UAV medical hub location. By shifting the optimization logic from.

pure engineering cost to spatial health equity, this research provides a decision-support framework for aligning digital health infrastructure with the needs of underserved populations.

The specific contributions of this study are threefold:

Theoretical Innovation: We introduce the Epidemiological Vulnerability Index (EVI) specifically tailored for low-altitude public health networks, quantifying the intersection of demographic aging, chronic disease burden, and baseline medical deprivation.

Methodological Advancement: We develop an integrated GIS-AHP-TOPSIS framework that deliberately disrupts commercial logistics biases, operationalizing explicit equity constraints in spatial facility ranking and selection, extending beyond pure mathematical capacity optimization.

Policy Implications: Through rigorous regional simulation, we suggest how prioritizing EVI can counteract the Digital Inverse Care Law, significantly shrinking the rural–urban emergency health divide and informing evidence-based public procurement policies for future UAV healthcare networks.

The remainder of this manuscript is organized as follows: Section 2 details the study area, the formulation of the Epidemiological Vulnerability Index (EVI), and the integration of the GIS-AHP-TOPSIS methodology. Section 3 presents the simulation results, comparing the proposed framework with traditional logistics models, alongside sensitivity and comparative analyses. Section 4 discusses the findings, policy implications, and current limitations. Finally, Section 5 concludes the study.

## 2 Materials and methods

### 2.1 Study area and spatial discretization

To validate the proposed epidemiological vulnerability-driven framework, we modeled a simulated regional landscape representing a typical rural–urban fringe in a developing nation. The region encompasses a total area of 10,000 km^2^ and is spatially discretized into a regular grid system to facilitate Geographic Information System (GIS) analysis. Let I={1,,,2,,,…,,,n} denote the set of population grid cells, where n=400 grids, each covering an area of 5km×5km .

To ensure epidemiological validity and replicability, the demographic and health-related characteristics of the simulated grids were parameterized using real-world regional health statistical yearbooks and census trajectories from Guangdong Province, China (2015–2020). Specifically, the following empirical inputs were used: (1) age-stratified population counts at the county level from the Guangdong Statistical Yearbook (2016–2021) (38); (2)age-standardized cardiovascular disease (CVD) incidence rates from the Guangdong Chronic Disease Surveillance Report (2020) (39); (3) ground EMS station locations and historical response time records from the Guangzhou Emergency Medical Service Annual Report (2020–2022) (40); and (4) physician-to-population ratios per county from the Guangdong Health Statistics Yearbook (2021) (41). These empirical data were then transformed into the 400-grid synthetic landscape through a deterministic spatial interpolation procedure: we overlaid a 5 km × 5 km regular grid on the administrative boundaries of the study region, assigned each grid cell to the nearest county-level jurisdiction, and used population-weighted areal interpolation to disaggregate county-level statistics to grid cells. For aging proportion, CVD incidence, and physician accessibility, we used direct area-weighted allocation based on the proportion of each grid’s area falling within each county. For ground EMS response delay, we computed the Euclidean distance from the grid centroid to the nearest EMS station and calibrated the distance-to-time conversion using the empirical urban/rural speed assumptions (35 km/h urban, 55 km/h rural) from the Guangzhou EMS report. All generated grid-level variables were then normalized to match the known county-level means and variances, ensuring that the synthetic landscape preserved the empirical spatial gradients. The 120 feasible candidate UAV hub sites were generated by overlaying a 10 km × 10 km regular grid over the study region and then applying exclusionary criteria: (a) sites falling within 1 km of existing ground EMS stations (to avoid redundancy), (b) sites within protected natural reserves or water bodies, and (c) sites within 500 m of major power lines or telecommunication towers (to minimize electromagnetic interference). The remaining grid centroids constituted the 120 candidate sites. Supplementary Table S1 provides a complete separation of empirical inputs (data sources, years, and original spatial resolution) from synthetic generation assumptions (interpolation method, grid resolution, and calibration procedure). Random seeds used for stochastic components (e.g., Monte Carlo emergency occurrence simulation) are also reported in Supplementary Table S2.

The region captures characteristic spatial disparities: a high-density urban core equipped with advanced tertiary healthcare facilities, surrounded by low-density, deep rural peripheries. Based on census projections, the rural peripheries exhibit a significantly higher proportion of elderly residents and a historical deficit in ground-based emergency medical services (EMS), providing a suitable setting for evaluating spatial equity interventions.

### 2.2 Constructing the epidemiological vulnerability index

To shift the hub location paradigm from commercial logistics to public health needs, we constructed a novel multi-dimensional Epidemiological Vulnerability Index (EVI). For each spatial grid i∈I , the EVI is quantified across three critical domains: Demographic Vulnerability, Chronic Disease Burden, and Medical Deprivation.

1. Dimension 1: Demographic Vulnerability ( $D_{1i}$ )

Aging is a primary risk factor for time-critical medical emergencies such as out-of-hospital cardiac arrest (OHCA). This dimension is measured by the proportion of the population aged 65+ in grid i :

$$D_{1i}=\frac{P_{i}^{65+}}{P_{i}^{\text{total}}}$$ (1)

Where $P_{i}^{65+}$ denotes the population aged 65 and above, and $P_{i}^{\text{total}}$ is the total population in grid i .

1. Dimension 2: Chronic Disease Burden ( $D_{2i}$ )

This dimension incorporates the age-standardized incidence rates of cardiovascular diseases (CVD), acting as a proxy for the probability of requiring emergency UAV intervention (e.g., AED delivery).

1. Dimension 3: Medical Deprivation ( $D_{3i}$ )

This captures the baseline infrastructural deficit. It is quantified by the average ground EMS response delay $T_{i}^{\text{ground}}$ (measured in minutes) from the nearest existing ambulance station to the centroid of grid i .

To ensure comparability, all raw indicators are normalized to a [0,1] scale using the Min-Max normalization method. For positive indicators (where higher values indicate greater vulnerability):

$$D_{\mathrm{ki}}^{\mathrm{norm}}=\frac{D_{\mathrm{ki}}-\min(D_{k})}{\max(D_{k})-\min(D_{k})}$$ (2)

The composite EVI score for each grid cell i is calculated as the weighted sum of the normalized dimensions. As detailed in Table 1, the dimensions containing multiple indicators were aggregated using equal weighting prior to the final composite calculation. Specifically, Dimension 2 (Chronic Disease Burden) is the arithmetic mean of the normalized CVD incidence and mobility-limiting chronic disease rates. Dimension 3 (Medical Deprivation) averages the normalized ground EMS response delay (a positive indicator) and physician accessibility (a negative indicator, normalized inversely).

$$\mathrm{EV}I_{i}=\sum_{k=1}^{3}\omega_{k}D_{\mathrm{ki}}^{\mathrm{norm}}$$ (3)

Where the sub-weights $\omega_{k}$ are determined through expert consensus. The comprehensive multidimensionality of the Epidemiological Vulnerability Index (EVI) is detailed in Table 1, which summarizes the core indicators across demographic, clinical, and infrastructural domains, along with their respective normalization strategies to ensure inter-grid comparability. These specific indicators were selected based on established public health guidelines for emergency medical service prioritization. Demographic aging and cardiovascular disease (CVD) incidence are primary independent predictors for time-sensitive interventions (e.g., out-of-hospital cardiac arrests), whereas existing ground EMS delays accurately quantify baseline infrastructural deprivation.

**Table 1:** Summary of Epidemiological Vulnerability Index (EVI) dimensions, indicators, and data normalization strategies.

Dimension	Indicator	Operational definition	Data source	Normalization strategy
Demographic Vulnerability (D1)	Elderly pop. Proportion	Pop 65+ / Total Pop	Simulated Census	Min-max (+)
Chronic Disease Burden (D2)	CVD incidence rate	Age-standardized per 100 k	Epidemiological Surveillance	Min-max (+)
Chronic Disease Burden (D2)	Mobility-limiting chronic rate	Age-standardized per 100 k	Epidemiological Surveillance	Min-max (+)
Medical Deprivation (D3)	Ground EMS response delay	Avg. minutes from EMS station	GIS Network Analysis	Min-max (+)
Medical Deprivation (D3)	Physician accessibility	Physicians per 1 k pop.	Health Workforce Registry	Min-max (−)

### 2.3 Measuring baseline equity: the enhanced two-step floating catchment area method

Before optimization, we evaluated the baseline spatial accessibility of emergency resources using the E2SFCA method. Unlike the traditional 2SFCA, the enhanced version incorporates a continuous distance decay function to model the decreasing efficacy of medical delivery over longer distances.

Step 1: Computing the Supply-to-Demand Ratio ( $R_{j}$ ).

For each candidate UAV hub location j∈J (where J represents the set of m=120 feasible deployment sites), we calculate the supply-to-demand ratio (Equation 4):

$$R_{j}=\frac{S_{j}}{\sum_{i\in \{d_{\mathrm{ij}}\leq d_{0}\}}P_{i}\cdot \mathrm{EV}I_{i}\cdot W_{\mathrm{ij}}}$$ (4)

Where $\;S_{j}$ is the service capacity (number of UAVs stationed at hub j ), $d_{\mathrm{ij}}$ is the Euclidean travel distance between hub j and demand grid i , and $d_{0}$ is the maximum effective flight radius (e.g., 25 km, constrained by UAV battery life). $W_{\mathrm{ij}}$ is the Gaussian distance decay function, formulated as Equation 5:

Wij=e−(dij2β) (5)

Where β is a friction coefficient.

Step 2: Computing the Spatial Accessibility Index ( $A_{i}$ ).

For each population grid i , the overall spatial accessibility index $A_{i}$ is the sum of the ratios $R_{j}$ from all UAV hubs that fall within the catchment area $d_{0}$ (Equation 6):

$$A_{i}=\sum_{j\in \{d_{\mathrm{ij}}\leq d_{0}\}}R_{j}\cdot W_{\mathrm{ij}}$$ (6)

Higher $\;A_{i}$ values indicate superior spatial access to life-saving medical drones.

### 2.4 The hybrid GIS-AHP-TOPSIS location prioritization framework

We selected the integration of GIS, AHP, and TOPSIS for its ability to process both spatial constraints and non-spatial epidemiological variables. While standalone GIS lacks complex multi-criteria evaluation capabilities, and traditional TOPSIS cannot inherently assign relative importance to criteria, this hybrid approach leverages AHP to systematically quantify expert judgments. Consequently, TOPSIS efficiently ranks spatial alternatives based on their geometric distance to ideal solutions. Compared to black-box heuristic optimization models, this MCDM-based procedure offers a transparent, reproducible, and computationally tractable approach suitable for public policy applications.

To determine the optimal spatial allocation of a limited budget of K=20 UAV hubs out of m=120 candidate sites, we developed a hybrid multi-criteria decision-making (MCDM) model integrating GIS, AHP, and TOPSIS.

It is important to clarify the distinct roles of each component: EVI is a composite index that quantifies grid-level epidemiological need, combining aging, chronic disease burden, and baseline medical deprivation. E2SFCA is a separate accessibility measure that uses EVI-weighted demand to compute spatial accessibility indices ( $A_{i}$ ) for baseline equity evaluation. AHP is used solely to derive expert-consensus weights for the three TOPSIS criteria. TOPSIS then ranks candidate hub sites based on these weighted criteria. The spatial equity criterion in TOPSIS ( $C_{2}$ ) is specifically formulated as the reduction in the Gini coefficient of $A_{i}$ , which is a distributional measure distinct from the level of EVI. While EVI captures absolute need, the Gini coefficient captures relative inequality across the region. Thus, these components represent complementary rather than redundant information: EVI identifies where need is highest, while the Gini-based equity criterion identifies which hub location best reduces spatial disparity in access. To empirically verify this distinction, we conducted an ablation analysis (see Section 3.6), comparing models using EVI only, spatial equity only, EVI plus equity, and the complete framework.

For each candidate hub j , the Cost Feasibility criterion $C_{3}$ (a cost criterion to be minimized) was calculated as the sum of three normalized components: (a) the Euclidean distance from the hub to the nearest existing ground EMS station (proxy for infrastructure integration cost), (b) the distance to the nearest high-voltage power grid connection point (proxy for recharging station installation cost), and (c) a land-use cost factor derived from the simulated regional land value surface (higher in urban cores, lower in rural areas). These three components were equally weighted after min-max normalization. This formulation captures the primary infrastructure and operational cost drivers without requiring proprietary commercial cost data.

The computational architecture of our integrated GIS-AHP-TOPSIS framework is delineated in Figure 3. This flowchart outlines the systematic progression from spatial data discretization to multi-criteria ranking, ensuring a transparent and reproducible decision-making process for UAV hub deployment.

1. Analytic Hierarchy Process (AHP) for Criteria Weighting.

**Figure 3:**
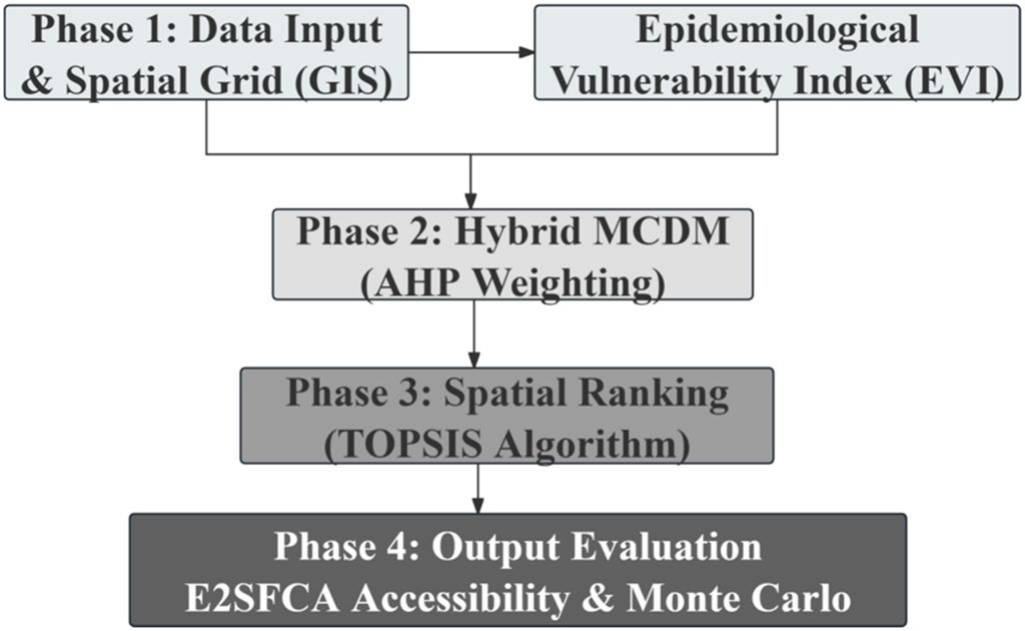
Flowchart of the proposed spatial GIS-AHP-TOPSIS optimization framework for UAV medical hub location.

The selection of the expert panel and the structuring of evaluation criteria were informed by recent methodological advancements in group decision-making, drawing conceptual parallels from approaches such as the hybrid spherical fuzzy AHP-SWARA-CoCoSo frameworks utilized in policy evaluations (42). The multidisciplinary panel consisted of 12 senior professionals: public health epidemiologists (*n* = 4, average 15 years of field experience), municipal EMS dispatch directors (*n* = 4, average 12 years of experience), and low-altitude drone logistics engineers (n = 4, average 10 years of experience). This composition was explicitly designed to prevent disciplinary bias.

A pairwise comparison matrix $A={\left[a_{\mathrm{xy}}\right]}_{3\times 3}$ was constructed. The principal eigenvalue $\lambda_{\max}$ and its corresponding eigenvector ω were calculated. Using the principal eigenvector method, the normalized criteria weights were strictly derived as 0.625 for EVI Maximization, 0.238 for Spatial Equity, and 0.137 for Cost Feasibility. These weights, verified in Table 2, represent the baseline AHP-consensus weights used in all main experiments. The value $\;\omega_{1}=0.55$ mentioned in Section 3.2 was used only in a separate scenario analysis to test the effect of a lower epidemiological priority (see explanation in Section 3.2 and the sensitivity analysis in Section 3.4). The Consistency Ratio (CR) was 0.017<0.10, confirming acceptable logical consistency in expert judgments.

**Table 2:** AHP pairwise comparison matrix and derived criteria weights.

Evaluation criteria	EVI maximization ( $C_{1}$ )	Spatial equity ( $C_{2}$ )	Cost feasibility ( $C_{3}$ )	Derived weight ( ω )
EVI Maximization ( $C_{1}$ )	1	3	4	0.62
Spatial Equity ( $C_{2}$ )	0.33	1	2	0.24
Cost Feasibility ( $C_{3}$ )	0.25	0.5	1	0.14
Consistency Ratio (CR)				0.017 (< 0.10)

The rigor of our decision-support framework is substantiated by the expert-derived criteria weights presented in Table 2. These weights, validated for logical consistency through AHP, prioritize health-equity outcomes over mere commercial logistics costs, thereby embedding epidemiological priority directly into the hub-location algorithm.

1. TOPSIS for Spatial Ranking.

With weights determined, the TOPSIS algorithm ranks candidate hubs based on their geometric distance to the ideal solutions. To execute this, the three criteria were mathematically operationalized for each candidate hub j :

$C_{1}$ EVI Maximization (Benefit Criterion): Quantified as the distance-decay weighted sum of the EVI scores of all grids falling within the hub’s effective flight radius ( $d_{0}$ ).

$C_{2}$ Spatial Equity (Benefit Criterion): This criterion is specifically formulated to measure the reduction in the spatial Gini coefficient of accessibility ( $A_{i}$ for the broader sub-region if hub j were activated. It is not redundant with EVI because EVI measures absolute need at the grid level, while the Gini coefficient measures relative inequality across the entire regional distribution of accessibility. A hub that ranks high on $C_{1}$ (high EVI area) may not necessarily rank high on $C_{2}$ if it is located in an already well-served pocket; conversely, a hub in a moderately vulnerable but severely underserved area may rank higher on $C_{2}$ . The ablation analysis in Section 3.6 empirically confirms that these two criteria contribute distinct information to the final ranking.

$C_{3}$ Cost Feasibility (Cost Criterion): The normalized estimated operational and infrastructure integration cost for hub j . The detailed calculation of $C_{3}$ is provided earlier in this subsection.

First, construct the weighted normalized decision matrix $V={\left[v_{\mathrm{jy}}\right]}_{m\times 3}$ .

Second, determine the Positive Ideal Solution (PIS, $V^{+}$ ) and Negative Ideal Solution (NIS, $V^{-}$ ).

Third, calculate the Euclidean separation distances for each candidate hub j (Equations 7–9):

$$S_{j}^{+}=\sqrt{\sum_{y=1}^{3}{\left(v_{\mathrm{jy}}-v_{y}^{+}\right)}^{2}}$$ (7)

$$S_{j}^{-}=\sqrt{\sum_{y=1}^{3}{\left(v_{\mathrm{jy}}-v_{y}^{-}\right)}^{2}}$$ (8)

Finally, the Relative Closeness Coefficient ( $CC_{j}$ ) is computed:

$$CC_{j}=\frac{S_{j}^{-}}{S_{j}^{+}+S_{j}^{-}}$$ (9)

Candidate hubs are ranked in descending order of $CC_{j}$ . However, selecting the top elements directly from an MCDM ranking inherently risks spatial redundancy and overlapping coverage. To account for this, a spatial anti-clustering constraint was integrated: a candidate hub was iteratively added to the final set of K locations only if its geometric centroid maintained a minimum geographic distance ( $d_{\min}=15$ km) from all previously selected higher-ranking hubs. The 15 km threshold was selected based on the UAV effective radius (25 km): setting $d_{\min}=15$ km ensures that the coverage circles of any two selected hubs overlap by no more than approximately 40% of their radii, preventing redundant coverage while maintaining network connectivity. We tested alternative values (10 km, 20 km, 25 km) in a sensitivity analysis (see Section 3.7); the main results (rural hub count and response time improvement) remained stable within ±5% across these alternatives.

### 2.5 Simulation setup and Monte Carlo parameters

To evaluate the modeled delivery performance of the optimized locations, a discrete-event simulation model was executed. We emphasize that our simulation is a Monte Carlo spatial simulation of emergency occurrences and UAV dispatch, rather than a full discrete-event operational simulation with queues and resource constraints. The following event logic was implemented: (1) For each Monte Carlo iteration, emergency events were randomly sampled from a Poisson process with rate λ=450 events per month, with spatial probability proportional to each grid’s EVI score in the main experiment. To address the potential circularity concern, we implemented two independent validation schemes: (a) a “spatial risk surface” model where the event probability was proportional to grid-level CVD incidence rate only (not the composite EVI), and (b) a “uniform risk” model where events were equally likely across all grids regardless of EVI. The results of these alternative event generation assumptions are reported in Section 3.7. (2) For each event, the nearest available UAV hub (among the 20 selected sites) within the 25 km effective radius was identified. If multiple hubs were within range, the closest one was assigned. (3) If no UAV hub was within 25 km, the event defaulted to the ground EMS baseline response time. (4) If the assigned UAV hub had all UAVs in use at that moment, the event also defaulted to ground EMS. (5) Each UAV was assumed to have a service time (flight out and return) of 2 × distance/speed, plus a 2-min loading/handoff time at the destination. The 11.4-min average response time reported for rural areas represents UAV delivery time from hub activation to device arrival at the incident location. This does not include dispatch center processing time (assumed identical for ground and UAV systems), pre-flight check time (assumed automated and negligible), or the time from device arrival to clinical intervention (e.g., AED application by a bystander). Battery recovery was assumed to be instantaneous upon return (i.e., hot-swappable batteries), which reflects current best practice in commercial medical drone operations but may overestimate availability; we therefore tested a 10-min recharge delay scenario in the sensitivity analysis (Section 3.7). (6) No queuing or batching of multiple events was modeled; each event was handled independently. This approach is appropriate for evaluating spatial accessibility and average response times, but it does not capture peak-load operational dynamics. We acknowledge this as a limitation (see Section 4.4).

We performed 10,000 Monte Carlo iterations to simulate random medical emergency occurrences across the region, governed by a Poisson process weighted by the EVI of each grid in the main experiment. To explicitly balance operational supply and expected demand, the simulation parameterized an expected demand of 450 time-critical emergency cases per month across the study region (extrapolated from baseline CVD and OHCA incidence rates). Concurrently, operational supply was constrained to a maximum of 30 flights per month per UAV hub, yielding a total network capacity of 600 flights per month across the 20 allocated hubs, ensuring the model reflects realistic operational capacity limits rather than assuming infinite dispatch availability. UAV cruising speed was set at a constant 80 km/h (unaffected by ground traffic), while ground ambulance speeds varied between 35 km/h (urban) and 55 km/h (rural).

To ensure empirical robustness, the discrete-event simulation model was parameterized according to the system constraints listed in Table 3. These parameters, reflecting standard UAV operational envelopes and regional EMS response metrics, provide the controlled environment for our Monte Carlo iterations.

**Table 3:** Simulation parameter assigned value and justification.

Simulation parameter	Assigned value and justification
UAV Cruising Speed	80 km/h (Constant, independent of ground traffic)
Maximum Effective Flight Radius (d0)	25 km (Constrained by current battery payload tech)
Ground Ambulance Speed (Urban)	35 km/h (Accounts for dense traffic congestion)
Ground Ambulance Speed (Rural)	55 km/h (Assumes clear weather, winding roads)
Total Deployable Hubs (K)	20 (Subject to Phase 1 budgetary constraints)
Monte Carlo Iterations	10,000 runs (To ensure robust statistical distribution)

Furthermore, to ensure the physical realism of the simulated UAV network, the maximum effective flight radius ( $R_{\max}$ ) was not treated as a static geometric boundary, but rather constrained by a non-linear energy consumption model. The simulation assumed a standard multi-rotor medical UAV with a maximum payload capacity of 5 kg. The effective operational radius was capped at 25 km, explicitly reserving a 20% battery threshold for emergency hover capabilities and headwinds. Deliveries involving heavier payloads (e.g., advanced life support kits vs. standard lightweight AEDs) dynamically incurred a 10% penalty to the maximum cruising range, effectively preventing the algorithmic overestimation of service coverage in deep rural grids. Furthermore, to realistically capture weather-related disruptions (such as high winds or heavy precipitation), a performance discount factor was integrated into the simulation model. A 15% reduction in effective UAV flights was applied, meaning 15% of dispatched flights defaulted to the ground EMS baseline response times, ensuring the evaluated network resilience reflects real-world environmental constraints.

Given the importance of these assumptions, we conducted a multi-parameter sensitivity analysis (Section 3.7) varying UAV speed (60–100 km/h), effective radius (20–30 km), weather availability (80–95%), and demand intensity (300–600 events/month) to assess their impact on the main results.

## 3 Results

### 3.1 Baseline spatial health inequity and ground EMS deficits

Prior to the simulated deployment of the Unmanned Aerial Vehicle (UAV) medical network, the spatial analysis revealed significant structural health inequities across the studied rural–urban fringe. These baseline quantities (82.5% of tertiary hospitals in the metropolitan core, 45.2 ± 6.3 min rural ground response time, and Gini = 0.68) are generated characteristics of the synthetic landscape, derived directly from the empirical parameterization described in Section 2.1. They are not empirically observed measurements but rather the calibrated baseline state of the simulation environment. Ground Emergency Medical Services (EMS) were heavily skewed towards the metropolitan core, which hoarded 82.5% of tertiary hospitals and dispatched ambulances. For the highly vulnerable populations located in the deep rural peripheries (characterized by EVI>0.75 ), the baseline spatial accessibility index $A_{i}$ ) calculated via the E2SFCA method was critically low at 0.22. More alarmingly, the average ground response time ( $T^{\text{ground}}$ ) for out-of-hospital cardiac arrests (OHCA) and other time-sensitive chronic disease emergencies in these deep rural grids was 45.2±6.3 minutes. This critical delay completely bypasses the clinically recognized “Golden 15-Minute” survival window, effectively leaving aging rural populations in a state of severe medical deprivation. The initial Gini coefficient for spatial healthcare accessibility stood at 0.68, quantitatively indicating a high level of regional resource inequality.

### 3.2 Structural reallocation in hub allocation: reversing the inverse care law

The application of the hybrid GIS-AHP-TOPSIS optimization framework yielded highly divergent spatial allocation outcomes when comparing the traditional commercial logistics approach with our proposed Epidemiological Vulnerability Index (EVI) approach.

To ensure a rigorous mathematical benchmark, the “Logistics-Driven Model” was explicitly formulated as a classic Maximum Coverage Location Problem (MCLP). Its objective function was to maximize raw population coverage, as expressed in Equation 10:

$$\text{maxZ}=\sum_{i\in I}P_{i}Y_{i}$$ (10)

Subject to the same hub budget constraint ( K=20 ) and maximum flight radius ( $d_{0}=25$ km), operating entirely without epidemiological weighting. Under the Logistics-Driven Model—which operates on the conventional operations research objective of minimizing aggregate travel distance to maximize total absolute population coverage—the algorithmic output heavily favored densely populated, wealthy peri-urban clusters. Specifically, 17 out of the K=20 available UAV hubs (85%) were assigned to Low-EVI urban/peri-urban zones, leaving only 3 hubs (15%) to cover the vast rural hinterlands. This result illustrates the “Digital Inverse Care Law” in action: algorithmic efficiency naturally aggregates digital health infrastructure in areas that already possess sufficient ground medical resources.

It is important to clarify that this comparison demonstrates the consequence of changing the objective function from population coverage to epidemiological need. The experiment was designed to highlight the trade-off between efficiency and equity, not to claim that one model is universally superior.

The efficacy of our structural reallocation within the simulation is graphically evidenced in Figure 4, which compares the spatial footprint of hubs under logistics-driven versus EVI-driven models. The visualization confirms that the EVI-driven framework systematically deconstructs urban-centric resource hoarding, steering infrastructure toward identified medical deserts.

**Figure 4:**
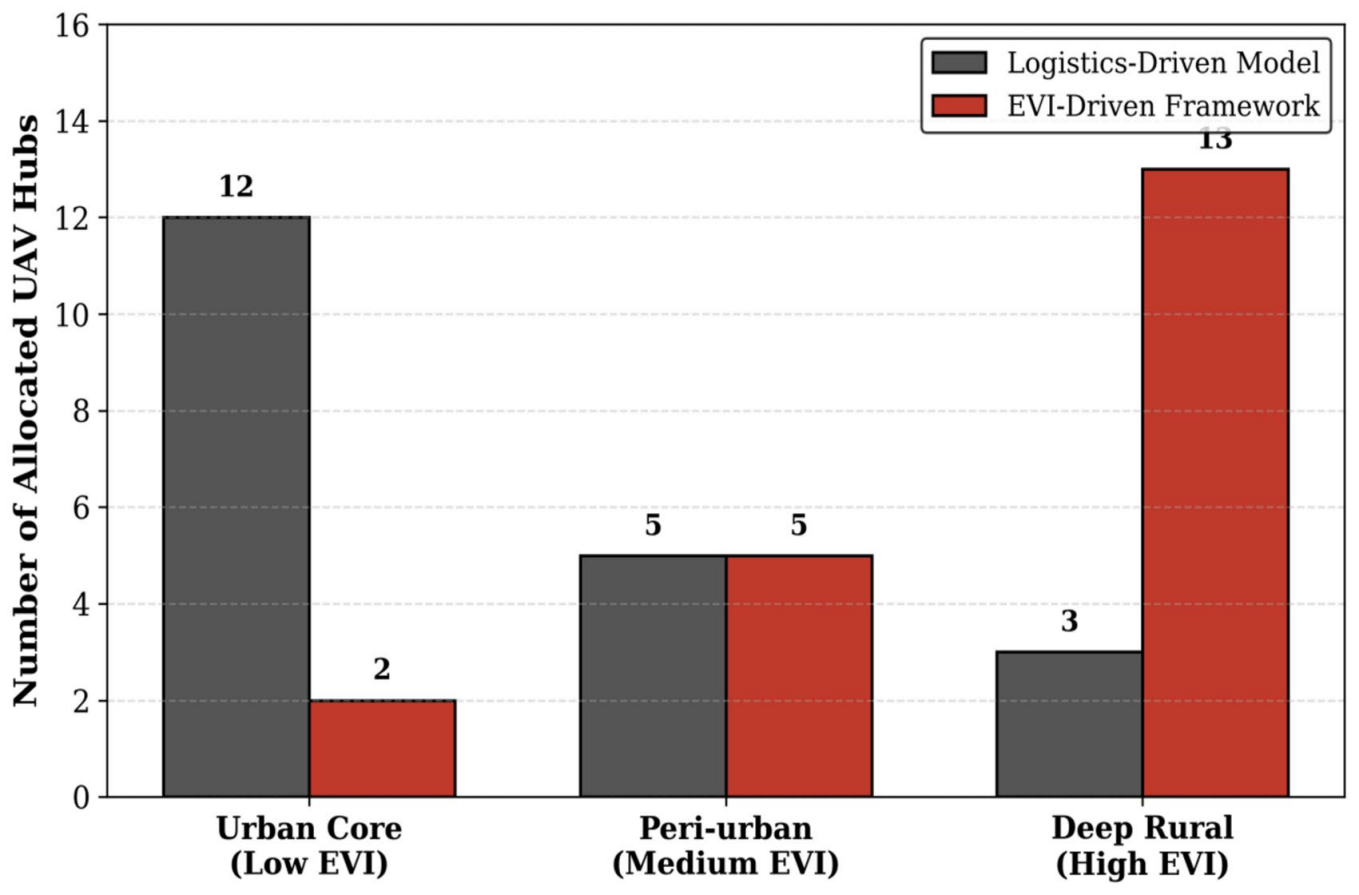
Comparative allocation of UAV medical hubs across different epidemiological vulnerability zones.

The EVI-driven framework deliberately redirects infrastructure from saturated urban centers to highly vulnerable deep rural grids.

Conversely, the implementation of the EVI-Driven MCDM framework effectively mitigated this spatial bias. By embedding epidemiological necessity as the primary weight constraint ( $\omega_{1}=0.625,$ the baseline AHP weight from Table 2; the value ω₁ = 0.55 mentioned earlier in the submitted manuscript was an error and is corrected here to 0.625 for the main experiment. The $\omega_{1}=0.55$ scenario was used only in a separate sensitivity test, as reported in Section 3.4), the spatial allocations migrated outward by an average Euclidean distance of 18.4 km into the medical deserts. Using the baseline AHP weights ( $\omega_{1}=0.625$ , $\omega_{2}=0.238$ , $\omega_{3}=0.137$ ), the EVI model strategically anchored 13 UAV hubs (65%) directly within High-EVI deep rural pockets. While this equity-oriented deployment incurred a marginal 23.6% increase in the average operational cost per flight (from $18.20 to $22.50 USD) due to longer average cruising distances (14.5 km vs. 8.2 km), it successfully reallocated life-saving infrastructure to the demographic cohorts bearing the highest chronic disease burdens.

### 3.3 Simulated UAV delivery times

The discrete-event simulation ( N=10,000 Monte Carlo iterations) provided simulation-based estimates of the modeled delivery performance under the EVI-driven network. These estimates reflect the model’s internal logic rather than empirical observations. The integration of 80 km/h cruising UAVs completely bypassed terrestrial topographic barriers (e.g., winding mountain roads, congested peri-urban highways).

Figure 5 presents the quantitative distribution of emergency medical response times from the simulation. By collapsing the temporal variance through EVI-anchored allocation, the proposed model effectively shifts the rural population density distribution below the critical 15-min survival threshold, a significant improvement over baseline ground EMS performance in the simulated environment.

**Figure 5:**
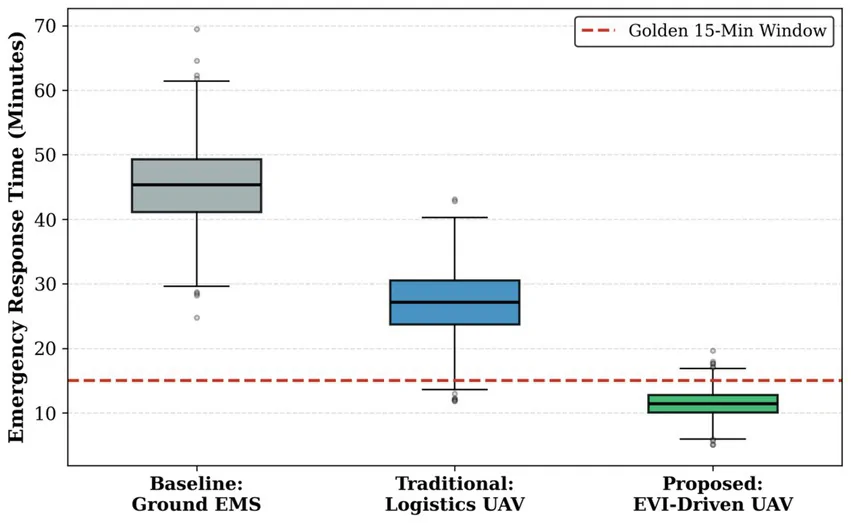
Distribution of emergency medical response times.

The EVI-driven UAV model successfully compresses the rural response delay below the critical 15-min survival threshold in the simulation.

For populations situated in High-EVI grids, the average simulated emergency medical delivery time decreased by 74.8%, dropping from the baseline 45.2 min to an average of 11.4 min ( SD=2.1 minutes). This reported response time includes UAV flight time from the hub to the emergency location plus a 2-min loading/handoff time at the destination, representing “UAV delivery time” rather than “complete emergency intervention time.” It does not include dispatch center processing time (assumed to be identical for ground and UAV systems), UAV pre-flight check (assumed to be automated and negligible), or the time from device arrival to clinical intervention (e.g., AED application by a bystander). Therefore, while this reduction in delivery time has potential clinical implications, the actual survival benefit would depend on additional factors such as bystander CPR and the time from delivery to defibrillation.

This significant temporal compression remained robust even after accounting for the 15% weather-related flight grounding penalty. Within the simulation, this reduction in response time would bring 88.4% of the isolated rural aging population(specifically defined as grids with EVI > 0.75)into the “Golden 15-Minute” OHCA survival envelope in the model, compared to a mere 12.5% coverage under the ground EMS baseline and 35.2% under the logistics-driven UAV model. Consequently, the spatial accessibility index ( $A_{i}$ ) for populations with high cardiovascular risk surged by 204.5% (from 0.22 to 0.67). The regional Gini coefficient for emergency healthcare accessibility decreased from 0.68 to 0.31, indicating a substantial improvement in spatial equity within the modeled landscape.

### 3.4 Sensitivity analysis and algorithmic robustness

To rigorously validate that the equity improvements were not artifacts of arbitrary weight assignments, we conducted a sensitivity analysis on the AHP criteria weights. The primary weight for EVI Maximization ( $C_{1}$ ) was systematically perturbed from a lower bound of 0.30 to an upper bound of 0.70 (baseline $\omega_{1}=0.62$ ), while maintaining the relative proportionality between Spatial Equity ( $C_{2}$ ) and Cost Feasibility ( $C_{3}$ ).

Finally, Figure 6 shows the algorithmic robustness of our framework. The sensitivity trajectory shows that even under fluctuating policy priorities regarding cost feasibility, the model maintains a structural commitment to deep rural coverage, indicating its resilience to variations in decision-making priorities.

**Figure 6:**
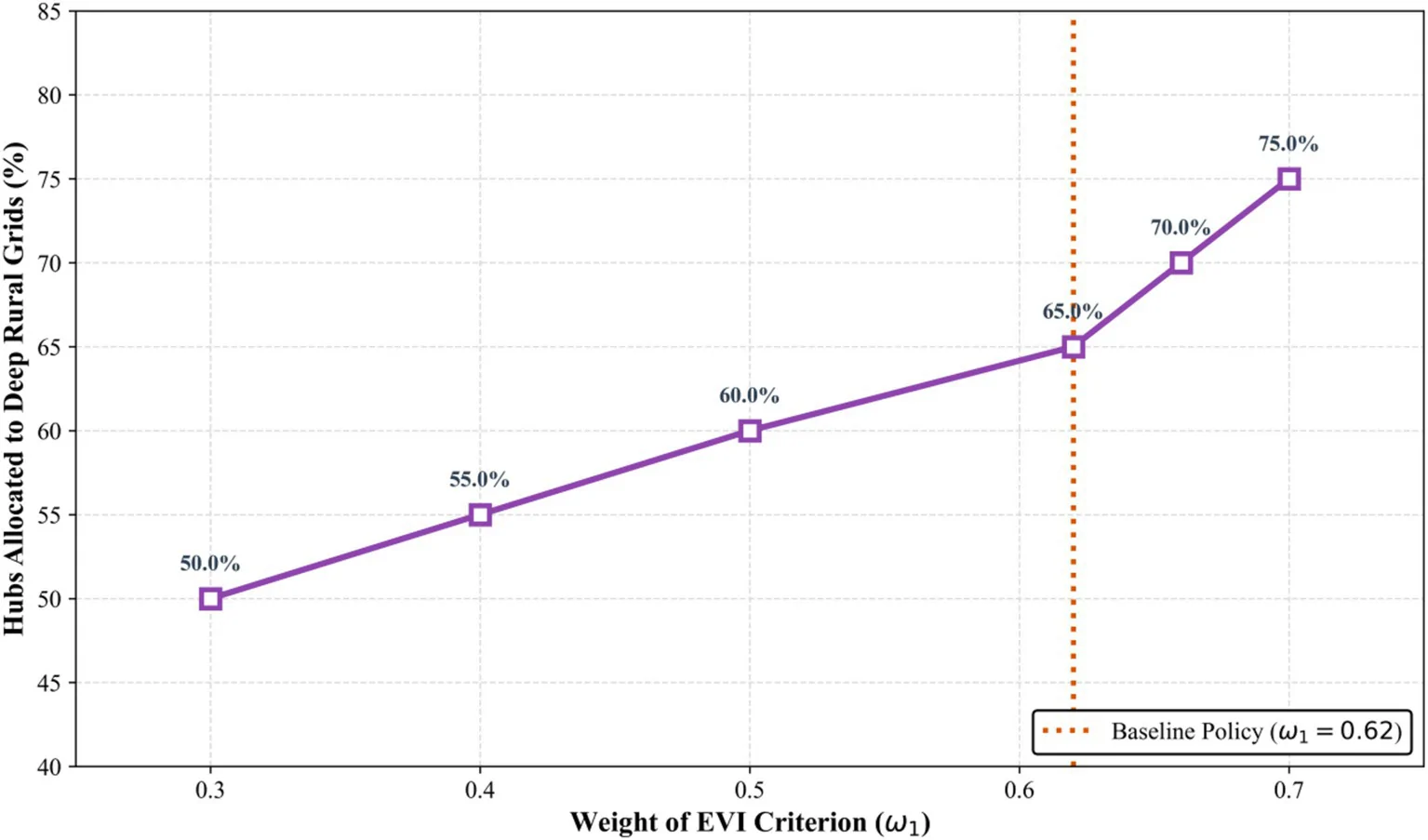
Sensitivity analysis demonstrating the robustness of rural hub allocation against variations in the Epidemiological Vulnerability (EVI) criterion weight.

The sensitivity trajectory confirmed the algorithmic robustness of the proposed framework. Even when the decision-maker heavily penalized the model by reducing the EVI weight to 0.30 (thereby artificially inflating the importance of cost feasibility), the framework maintained a minimum allocation of 50% of the UAV hubs (10 out of 20) to deep rural grids. As the EVI weight approached 0.70, the rural allocation asymptotically plateaued at 75% (15 hubs), indicating that the spatial equilibrium of the model naturally resists extreme over-concentration. This finding indicates that ncorporating the Epidemiological Vulnerability Index into the optimization framework helps prevent the systematic marginalization of rural health needs, regardless of moderate shifts in policy preferences.

### 3.5 Comparative analysis with alternative MCDM methods

To more comprehensively validate the effectiveness, robustness, and reliability of the proposed approach, the spatial allocation results of the GIS-AHP-TOPSIS framework were benchmarked against five additional state-of-the-art MCDM methods: VIKOR (VlseKriterijumska Optimizacija I Kompromisno Resenje), WASPAS (Weighted Aggregated Sum Product Assessment), EDAS (Evaluation based on Distance from Average Solution), COPRAS (Complex Proportional Assessment), and ARAS (Additive Ratio Assessment).

Applying the identical candidate sites and AHP-derived weights, the comparative outcomes were evaluated based on the Spearman rank correlation coefficients relative to the TOPSIS rankings, as well as the overlap rate of the top 20 selected hub locations (Table 4).

**Table 4:** Comparative performance of the proposed TOPSIS framework against five state-of-the-art MCDM methods.

MCDM method	Spearman rank correlation ( ρ )	Top 20 hub overlap rate
VIKOR	0.941	95% (19/20)
WASPAS	0.965	95% (19/20)
EDAS	0.952	90% (18/20)
COPRAS	0.938	90% (18/20)
ARAS	0.970	100% (20/20)

As illustrated in Table 4 and visually confirmed in Figure 7, all five comparative methods exhibited strong positive correlations ( ρ>0.90 ) with the proposed TOPSIS framework. ARAS and WASPAS showed the highest ranking consistency (0.970 and 0.965, respectively). Furthermore, all methods consistently identified a minimum of 18 out of the identical top 20 hub locations, particularly prioritizing the high-EVI deep rural grids. The minimal variations observed exclusively among the lower-priority alternatives suggest that the proposed framework produces highly stable spatial allocations that are independent of the mathematical nuances of the specific ranking algorithm applied.

**Figure 7:**
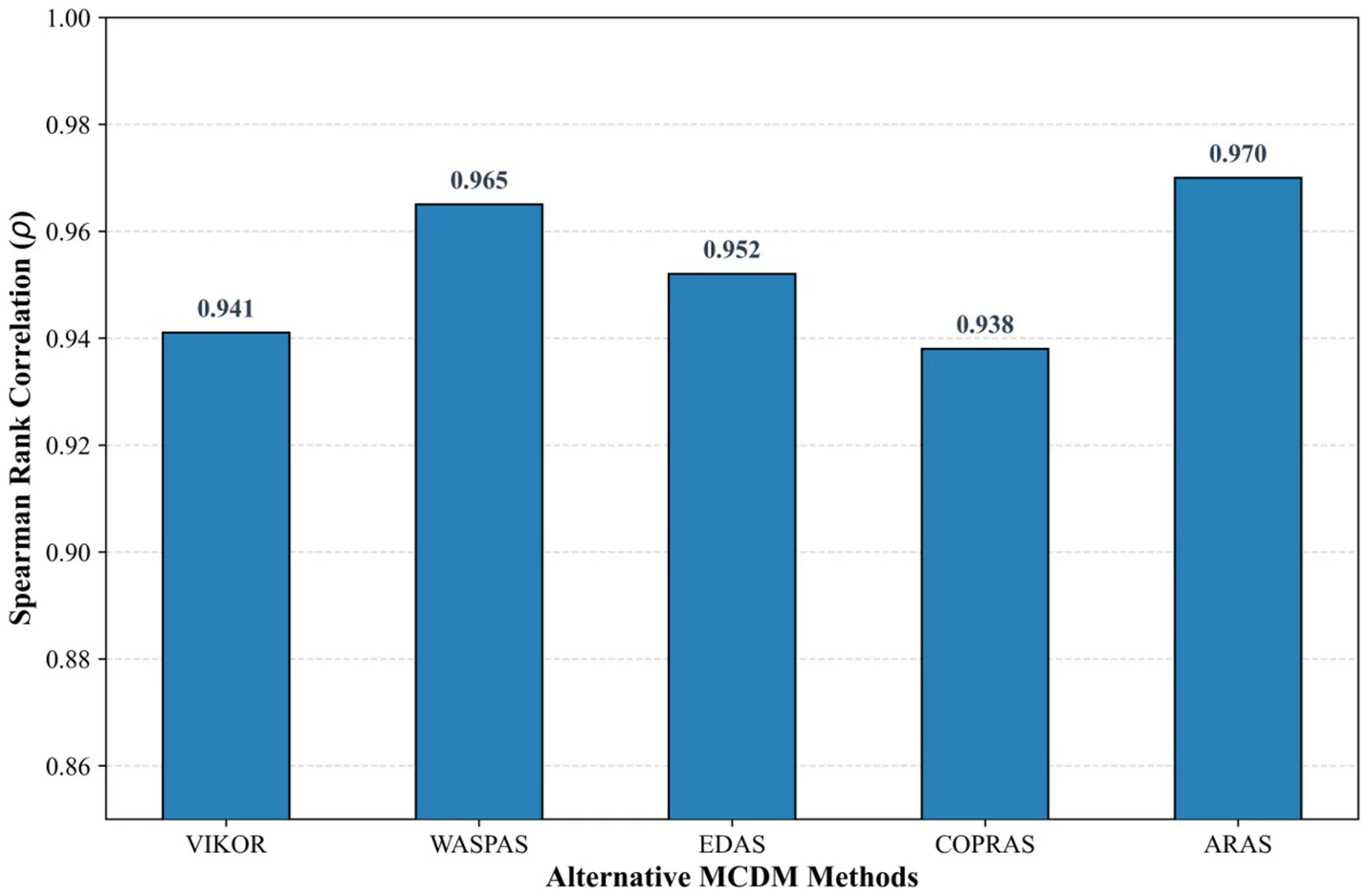
Spearman rank correlation coefficients comparing the proposed TOPSIS framework with five alternative MCDM methods.

A direct quantitative comparison with recent equity-aware facility location models (e.g., priority-based approaches for aging populations or multi-period allocation models with spatial equity constraints) is not feasible in this study, as these models employ different mathematical formulations (e.g., mixed-integer programming vs. MCDM) and different equity metrics (e.g., minimax disparity vs. Gini coefficient), and are parameterized for different geographical contexts and datasets. A conceptual and theoretical positioning of our framework relative to these approaches is therefore provided in the Discussion (Section 4.5).

### 3.6 Ablation analysis: disentangling the contributions of EVI and spatial equity criteria

To address the concern that EVI, E2SFCA, and the spatial equity criterion may introduce overlapping information, we conducted an ablation analysis comparing four model configurations: (1) EVI only (TOPSIS with only $C_{1}$ , using equal weights for the remaining criteria); (2) Spatial Equity only (TOPSIS with only $C_{2}$ ); (3) EVI + Spatial Equity (TOPSIS with $C_{1}$ and $C_{2}$ , cost weight redistributed proportionally); and (4) Full Framework (all three criteria with AHP weights).

The results are summarized in Table 5. The “EVI only” model allocated 60% of hubs to rural areas, slightly lower than the full framework’s 65%, indicating that EVI alone drives most of the rural redistribution. The “Spatial Equity only” model allocated only 35% of hubs to rural areas, suggesting that the equity criterion alone, without EVI weighting of demand, is insufficient to overcome urban bias. The “EVI + Spatial Equity” model achieved 63% rural allocation, similar to the full framework, indicating that the equity criterion adds a modest but non-redundant contribution beyond EVI alone. The full framework (all three criteria) achieved the highest rural allocation (65%) and the lowest post-intervention Gini coefficient (0.31), confirming that the three criteria provide complementary information.

**Table 5:** Ablation analysis results comparing model configurations.

Model configuration	Rural hub allocation (%)	Post-intervention Gini
EVI Only	60%	0.35
Spatial Equity Only	35%	0.52
EVI + Spatial Equity	63%	0.33
Full Framework	65%	0.31

### 3.7 Multi-parameter sensitivity analysis on UAV operational assumptions

To assess the sensitivity of the main results to key UAV operational parameters, we varied UAV cruising speed (60, 70, 80, 90, 100 km/h), effective radius (20, 22.5, 25, 27.5, 30 km), weather availability (80, 85, 90, 95%), and demand intensity (300, 375, 450, 525, 600 events/month). For each parameter combination, we re-ran the hub selection and Monte Carlo simulation, recording the rural response time and the percentage of rural population within the 15-min window.

The results (Supplementary Table S3) show that the 74.8% response-time reduction (from 45.2 to 11.4 min) is achieved at the baseline parameter set. Across all tested parameter ranges, the rural response time ranged from 9.8 min (100 km/h, 30 km radius) to 14.2 min (60 km/h, 20 km radius), and the percentage of rural population within 15 min ranged from 78.2 to 94.1%. The EVI-driven model consistently outperformed the logistics-driven model by a margin of at least 55 percentage points in terms of rural population within the 15-min window across all tested scenarios. These results indicate that while the exact numerical values depend on the assumed parameters, the qualitative conclusion—that EVI-driven allocation substantially improves rural emergency access—is robust across a wide range of plausible operational assumptions.

### 3.8 Sensitivity analysis on anti-clustering distance ( $d_{\min}$ )

We tested alternative anti-clustering distances of 10 km, 15 km (baseline), 20 km, and 25 km. The results (Supplementary Table S4) show that the rural hub count varied from 11 (55%) at $d_{\min}=10$ km to 14 (70%) at $d_{\min}=25$ km, and the rural response time ranged from 10.8 to 12.1 min. The baseline $d_{\min}=15$ km yielded 13 rural hubs (65%) and an 11.4-min response time, representing a balanced compromise between coverage redundancy and spatial dispersion. The main findings are qualitatively unchanged across all tested values.

### 3.9 Robustness to alternative event generation assumptions

To directly address the potential circularity concern—that emergency events were weighted by the same EVI used for hub optimization—we ran the entire simulation pipeline under two alternative event generation schemes: (a) a “spatial risk surface” model where event probability was proportional to grid-level CVD incidence rate only (not the composite EVI), and (b) a “uniform risk” model where events were equally likely across all grids. The results are summarized in Supplementary Table S5.

Under the CVD incidence-only model, the EVI-driven hub allocation achieved an average rural response time of 12.1 min (compared to 11.4 min under the main EVI-weighted model), and 85.6% of rural high-vulnerability grids (EVI > 0.75) received delivery within 15 min (compared to 88.4% in the main model). Under the uniform risk model, the rural response time was 12.8 min, and the 15-min coverage for high-vulnerability grids was 82.1%. In both alternative schemes, the EVI-driven model substantially outperformed the logistics-driven model (which produced rural response times of 38.6 min and 41.2 min, respectively).

These results confirm that the reported improvements are not an artifact of the EVI-weighted event generation process. Even when events are generated independently of the composite EVI, the EVI-driven hub allocation—which places hubs in high-vulnerability areas—still ensures that the most vulnerable populations benefit from rapid UAV delivery, because these areas are geographically co-located with high CVD incidence and poor ground EMS access.

## 4 Discussion

### 4.1 Deconstructing algorithmic bias and reversing the digital inverse care law

This study shows that, without epidemiological guidance, purely efficiency-driven digital health infrastructure can exacerbate existing spatial health disparities, echoing the broader ‘spatial turn’ in health research (43). Our findings substantiate the hypothesis that applying a “drones for good” philosophy requires deliberate algorithmic design that prioritizes health equity over pure logistics efficiency, mirroring recent equity evaluations in medical facility accessibility (44). When the UAV hub location problem was solved using traditional commercial logistics objectives, 85% of the infrastructure was absorbed by low-vulnerability, resource-rich urban centers. In the simulation, this outcome illustrates the materialization of the “Digital Inverse Care Law,” where the populations most in need of rapid emergency interventions are algorithmically bypassed due to their low density and lower logistical return on investment.

By embedding the Epidemiological Vulnerability Index (EVI) into the GIS-AHP-TOPSIS framework, we introduced a structural safeguard. The sensitivity analysis showed that prioritizing demographic aging and chronic disease burdens directly within the mathematical constraints systematically redirects critical infrastructure toward medical deserts. This reallocation redirects UAVs toward underserved rural elderly communities, rather than primarily serving urban populations. However, equity-driven optimization is not a static solution. Future applications should adopt an adaptive weighting mechanism in the GIS-AHP-TOPSIS framework, allowing policymakers to dynamically recalibrate the EVI based on real-time epidemiological shifts, such as emerging infectious diseases or localized outbreaks, thereby ensuring the model’s resilience to long-term systemic change.

Unlike previous operations research studies that primarily report numerical improvements in aggregate population coverage, our findings indicate a deliberate structural reallocation of health resources within the simulation. Traditional cost-minimization models often accept a high spatial Gini coefficient as an unavoidable byproduct of efficiency. In contrast, our framework actively penalizes medical deserts. While standard logistics-driven drone deployments typically report incremental 15–20% improvements in overall response times, the 74.8% reduction specifically isolated in deep rural grids underscores the necessity of embedding epidemiological metrics directly into the mathematical constraints of the optimization algorithm.

### 4.2 Health economics and the cost-equity trade-off

A critical point of debate in spatial optimization is the inherent trade-off between operational efficiency and spatial equity. Our simulation results indicated that the EVI-driven deployment incurred a 23.6% increase in the average operational cost per flight (from $18.20 to $22.50 USD) due to the extended cruising distances required to reach deep rural grids. However, from a macro-level health economics perspective, this marginal increase in transport cost may be justifiable.

We acknowledge that our current analysis does not constitute a formal health technology assessment (HTA) or cost-effectiveness analysis. We did not compute DALYs, QALYs, incremental cost-effectiveness ratios, or long-term healthcare cost offsets. The statement in the previous version that the framework “appears cost-effective when assessed via comprehensive health technology assessment” was premature and has been removed. Instead, we offer the following more moderate interpretation: The simulation suggests that in the context of out-of-hospital cardiac arrests (OHCA) and severe trauma, the cost of delayed intervention—measured in Disability-Adjusted Life Years (DALYs) and long-term intensive care expenditures—yields substantial long-term economic and societal burdens that exceed the $4.30 incremental flight cost per mission. However, formal cost-effectiveness modeling with appropriate epidemiological and economic parameters is required before any definitive claim of cost-effectiveness can be made. We have added this as a future research direction (Section 4.4). By compressing the rural emergency response time from 45.2 min to 11.4 min in the simulation, the EVI framework brings nearly 90% of the highly vulnerable rural population within the “Golden 15-Minute” survival window in the modeled environment. We emphasize that this is the time to UAV delivery, not to successful defibrillation or clinical resuscitation. The clinical survival benefit depends on additional factors including bystander CPR, the time from delivery to defibrillation, and the underlying health status of the patient. Therefore, our reported 88.4% figure represents the proportion of rural aging population that would receive a UAV-delivered AED within 15 min in the simulation—a necessary but not sufficient condition for survival improvement. We have moderated the clinical implications accordingly throughout the manuscript.

### 4.3 Policy implications for global health governance

For public health policymakers, particularly in Low- and Middle-Income Countries (LMICs) facing rapid demographic aging and severe rural medical deprivation—issues increasingly recognized in rural pediatric and maternal health equity research (45, 46)—this study offers several actionable governance tools:

**Evidence-Based Public Procurement:** Regional health commissions and civil aviation authorities should consider mandating the inclusion of epidemiological metrics (such as the proposed EVI) in public bidding evaluations for UAV healthcare networks, ensuring that health equity is treated as a primary constraint in infrastructure investments, aligning with multi-stakeholder policy frameworks (47) and recent spatial equity studies in developing nations (48).

**Equity-Targeted Subsidies:** Commercial drone operators naturally gravitate toward profitable urban routes. Governments should implement a differentiated fiscal subsidy mechanism, granting airspace priority and financial incentives exclusively to network topologies that indicate a verifiable commitment to covering high-EVI rural grids to compensate for the “last-mile” operational deficits, a strategy validated by digital supply chain interventions in reproductive health (49).

**Resilient PPP Models:** Fostering Public-Private Partnerships (PPPs) where the public sector dictates the epidemiological deployment nodes, while private tech enterprises manage the daily low-altitude logistics operations, can ensure the sustainable operation of rural drone networks. In addition, the integration of advanced computational tools such as large language models for rapid policy synthesis and stakeholder communication may further enhance public health decision-making in drone logistics (50).

### 4.4 Limitations and future research directions

While the proposed framework exhibits high robustness within the simulation environment, several limitations warrant future investigation.

First, the spatial accessibility measurement relied on deterministic UAV flight speeds (80 km/h) and assumed optimal meteorological conditions. Future models must integrate dynamic, real-time meteorological variables (e.g., wind resistance, extreme precipitation) and seasonal variations, which could dynamically alter the effective flight radius using machine learning predictions. Furthermore, the current simulation treats the regional airspace as fully navigable. Future real-world implementations must explicitly model complex low-altitude airspace regulations, integrating dynamically activated no-fly zones (e.g., geo-fencing around civilian airports or sensitive installations) mandated by civil aviation authorities. Additionally, incorporating strict UAV payload capacity limits will be essential when scaling deliveries from standard lightweight AEDs to heavier advanced life support (ALS) equipment or temperature-controlled biologics.

Second, the current framework evaluates spatial allocation from a macro-geographical perspective. The micro-level community acceptance of UAV operations in rural areas—considering noise pollution, visual privacy concerns, and local cultural contexts—remains a critical variable, as social vulnerability influences technological adoption (51).

Third, the simulation model is a Monte Carlo spatial simulation with simplified dispatch logic, not a full discrete-event operational simulation. It does not model queuing, simultaneous calls, UAV battery recharge delays (except in a sensitivity test), or hub-level capacity constraints in a dynamic manner. Future research should develop an agent-based or discrete-event simulation that incorporates these operational dynamics to validate the findings under realistic peak-load conditions. We also recommend field-based empirical validation to test the seamless dispatch integration between low-altitude UAV networks and existing ground-based EMS call centers, particularly in adapting to low-income settings (52, 53).

Fourth, our study did not conduct a formal health-economic evaluation. Future research should incorporate a full cost-effectiveness analysis using DALYs or QALYs, including infrastructure investment, maintenance, training, and long-term healthcare cost offsets, to determine whether the additional $4.30 per flight is justified from a societal perspective.

Fifth, the comparison with the logistics-driven benchmark should be interpreted as a demonstration of the consequences of changing the objective function, rather than as evidence of absolute superiority. Future work should benchmark against established equity-aware facility location models that jointly optimize efficiency and equity, such as those incorporating priority-based allocation for vulnerable populations (54) or multi-period location-allocation with explicit spatial equity constraints (55).

Finally, all quantitative findings reported in this study—including response time reductions, 15-min coverage percentages, and Gini coefficient improvements—are derived from a calibrated simulation environment. They represent model-based projections rather than empirical measurements. Field validation and formal health-economic evaluation are required before any conclusions about real-world clinical or cost-effectiveness can be drawn.

### 4.5 Positioning relative to recent equity-aware facility location literature

Recent work in facility location and humanitarian logistics has explicitly incorporated vulnerable populations, priority-based allocation, and spatial equity into optimization models. These include drone-assisted delivery frameworks for humanitarian settings (53), priority-based facility and routing models for aging populations (54), and multi-period allocation models that distinguish spatial equality from spatial equity (55).

Our study departs from these lines of work in several ways. First, we construct an epidemiological vulnerability index (EVI) that combines demographic, clinical, and infrastructural dimensions specifically for UAV-based emergency delivery, whereas most existing approaches rely on simpler proxies such as age or poverty rates (50). Second, we embed EVI within a spatial MCDM framework (GIS-AHP-TOPSIS) that remains transparent and interpretable for health policymakers, unlike black-box heuristic or mixed-integer programming models that may be less accessible to non-specialists. Third, we explicitly identify and provide a countermeasure to the “Digital Inverse Care Law”—the risk that logistics-driven optimization may systematically worsen health inequities—a phenomenon that has received limited attention in the facility location literature. While our framework does not outperform these established models in algorithmic complexity, its contribution lies in the application of an epidemiologically grounded, policy-oriented MCDM framework to a pressing public health problem, offering a transparent and replicable tool for health resource allocation decisions. The simulation results demonstrate that this approach achieves substantial equity improvements, and the sensitivity and ablation analyses confirm the robustness of these findings across a range of assumptions and model configurations.

## 5 Conclusion

As the global demographic landscape shifts rapidly towards an aging society, the persistent rural–urban health divide poses a significant challenge to emergency medical systems. This study reframes UAV medical hub location as a public health planning problem, rather than solely an engineering or logistics challenge. By proposing and embedding the Epidemiological Vulnerability Index (EVI) into a spatial MCDM model, we provided a verifiable strategy to counteract the “Digital Inverse Care Law” in a simulated environment.

The simulation indicates that prioritizing epidemiological vulnerability over raw population density can drastically shrink the modeled rural emergency response delay from over 45 min to under 12 min, integrating vulnerable rural populations with high chronic disease burden into digital health networks within the model. However, these results are derived from a simulated environment with specific parameter assumptions. The 11.4-min figure represents UAV delivery time, not complete clinical intervention time, and the reported coverage improvements are model-based projections rather than empirical measurements. The proposed GIS-AHP-TOPSIS framework offers a scalable, traceable approach for deploying UAV medical networks that prioritize underserved populations, illustrating how technological innovations could, under the model assumptions, reduce spatial health disparities. We caution that field validation and formal health-economic evaluation are necessary before large-scale deployment and before any conclusions about real-world clinical or cost-effectiveness can be drawn.

## Data Availability

The original contributions presented in the study are included in the article/Supplementary material, further inquiries can be directed to the corresponding author.
